# The application of the comet assay to assess the genotoxicity of environmental pollutants in the nematode *Caenorhabditis elegans*

**DOI:** 10.1016/j.etap.2016.06.020

**Published:** 2016-07

**Authors:** Soudabeh Imanikia, Francesca Galea, Eszter Nagy, David H. Phillips, Stephen R. Stürzenbaum, Volker M. Arlt

**Affiliations:** a Analytical and Environmental Sciences Division, MRC-PHE Centre for Environment and Health, King’s College London, London, United Kingdom; b NIHR Health Protection Research Unit in Health Impact of Environmental Hazards at King’s College London in partnership with Public Health England, London, United Kingdom

**Keywords:** Single cell gel electrophoresis assay, Comet assay, *C. elegans*, Benzo[*a*]pyrene, DNA repair

## Abstract

This study aimed to establish a protocol for cell dissociation from the nematode *Caenorhabditis elegans* (*C. elegans*) to assess the genotoxicity of the environmental pollutant benzo[*a*]pyrene (BaP) using the alkaline version of the single cell electrophoresis assay (comet assay). BaP genotoxicity was assessed in *C. elegans* (wild-type [WT]; N2, Bristol) after 48 h exposure (0–40 μM). Induction of comets by BaP was concentration-dependent up to 20 μM; comet% tail DNA was ∼30% at 20 μM BaP and ∼10% in controls. Similarly, BaP-induced DNA damage was evaluated in *C. elegans* mutant strains deficient in DNA repair. In *xpa-1* and *apn-1* mutants BaP-induced comet formation was diminished to WT background levels suggesting that the damage formed by BaP that is detected in the comet assay is not recognised in cells deficient in nucleotide and base excision repair, respectively. In summary, our study provides a protocol to evaluate DNA damage of environmental pollutants in whole nematodes using the comet assay.

## Highlights

• A protocol for the isolation of cells from *C. elegans* was established.
• The genotoxicity of benzo[a]pyrene (BaP) in *C. elegans* was assessed using the comet assay.
• Comet formation by BaP was dose-dependent.
• Both NER- and BER-related single-strand breaks contribute to comet formation by BaP.

## 1 Introduction

The single-cell gel electrophoresis assay (comet assay) is widely used for the detection of DNA damage and repair in a variety of cells *in vitro* and *in vivo* (Olive and Banath, 2006). DNA damage detected by the alkaline version of the comet assay includes single- and double-strand breaks and alkali-labile (*e.g*. apurinic) sites. The assay has the advantage of being a rapid, sensitive and relatively inexpensive method. It is not only commonly used in genotoxicity testing but it also has widespread applications in environmental biomonitoring and human population monitoring (Amaeze et al., 2015, Forchhammer et al., 2012).

The invertebrate nematode *Caenorhabditis elegans* (*C. elegans*) is a useful model organism for studying toxicogenomic responses to environmental pollutants at the molecular level as well as at the level of the organism (Polak et al., 2014, Steinberg et al., 2008). Due to the availability of the whole genome sequence, *C. elegans* has been subjected to gene expression studies (Steinberg et al., 2008). Further, *C. elegans* has been shown to be a useful alternative to mammalian models because mutant strains can be generated in which distinct genes are knocked out or are genetically modified (*e.g*. reporter gene assays) (Imanikia et al., 2015, Qabazard et al., 2013). Recently the mutational signatures of different chemotherapeutic drugs and environmental carcinogens in *C. elegans* were studied by whole genome sequencing (Meier et al., 2014).

Polycyclic aromatic hydrocarbons (PAHs) are products of incomplete combustion of fossil fuels and are present in the particulate phase of polluted air, diesel exhaust, and tobacco smoke (Phillips, 1999). Benzo[*a*]pyrene (BaP) is often studied as a model PAH and is listed as Group 1 human carcinogen by the International Agency for Research on Cancer (IARC). In mammalian cells BaP requires metabolic activation by cytochrome P450 enzymes to induce genotoxicity, *i.e*. formation of covalent DNA adducts [10-(deoxyguanosin-*N*^2^-yl)-7,8,9-trihydroxy-7,8,9,10-tetrahydrobenzo[*a*]pyrene (dG-*N*^2^-BPDE)] (Arlt et al., 2015, Krais et al., 2016, Wohak et al., 2016). Other pathways by which BaP can exert its genotoxic properties involve the formation of radical cations and *o*-quinones (Luch and Baird, 2005, Penning, 2014). However, little is known about the genotoxic potential of BaP in *C. elegans* (Leung et al., 2010, Neher and Sturzenbaum, 2006). End-points used to investigate the effects of BaP in *C. elegans* primarily focused on lethality, reproduction and behavioral responses (Dhawan et al., 1999). Comparison of these end-points showed that reproduction and movement were more sensitive than lethality in assessing BaP toxicity (Dhawan et al., 1999).

In the present study the comet assay was used to assess genotoxicity in *C. elegans*. For the first time we established a cell dissociation method from the whole worms to apply in the single-cell gel electrophoresis assay (comet assay) to determine the genotoxicity of BaP.

## 2 Material and methods

### 2.1 Carcinogens

Benzo[*a*]pyrene (BaP; CAS number 50-32-8; purity ≥96%) was obtained from Sigma-Aldrich.

### 2.2 *C. elegans* strains and maintenance

*C. elegans* strains were maintained at 20 °C on Nematode Growth Media (NGM) plates supplemented with *Escherichia coli* OP50 as the food source as described previously (Imanikia et al., 2015). N2 Bristol was used as the wild-type strain (WT) and obtained from the *Caenorhabditis* Genetics Center, University of Minnesota. The following deletion mutants were kindly provided by Prof. Anton Gartner, University of Dundee: *xpa-1(ok698)*, *xpf-1(tm2842)*, *apn-1(LS3678)* (Vallin et al., 2012) and *exo-3(ok3539)*. All mutants are null alleles and eliminate a sizable proportion of the respective open reading frames. Further information can be obtained at the National Bioresource Project for the Nematode and on www.wormbase.org. For each assay, nematodes were age-matched using an alkaline hypochlorite treatment to isolate the eggs (Imanikia et al., 2015). Thereafter, eggs were allowed to hatch overnight in M9 buffer and arrested at L1 stage. On the following day, age-synchronous L1 worms were transferred to NGM plates and utilized for the assays.

### 2.3 Exposure of *C. elegans* to BaP

For each exposure assay, a fresh culture of *E. coli* OP50 bacteria was mixed with the appropriate volume of the BaP stock solution to reach the designated final concentration. An equal amount (final concentration of 0.6%) of the solvent vehicle dimethysulfoxide (DMSO) was added to all bacterial cultures (including the untreated control). An aliquot of a freshly made mixture (bacteria + BaP [5–40 μM] or DMSO only) was seeded on NGM plates and incubated at 37 °C for 24 h. On the same day, gravid nematodes (wild-type and/or mutant strains) were age-synchronized by the alkaline-hypochlorite method (Imanikia et al., 2015), and eggs were left to hatch overnight. Thereafter, L1 larva were plated on 90-mm petri dishes that contained overnight grown bacteria (grown either with or without BaP, and grown either with or without DMSO); approximately 1200 L1 age-synchronized nematodes were added to each 90-mm petri dish. Worms were incubated for 42–48 h to reach the L4 stage for the purpose of cell isolation.

In initial experiments, OP50 growth was investigated over a period of 24 h after exposure to 20 and 40 μM of BaP. A fresh culture of OP50 was prepared by overnight incubation of a single colony of *E. coli* OP50 in a total of 15 mL LB broth. On the following day, the density of the overnight culture was determined by measuring the optical density (OD; *i.e*. light absorbance) at 595 nm. Next, bacterial density was adjusted to OD = 0.1 by diluting in fresh LB broth to start carcinogen exposure when bacteria were at the lag phase. For each designated concentration a bacterial suspension was prepared in a total volume of 15 mL in 50-mL tubes. Subsequently the OD at 595 nm was measured at 1, 2, 3, 4, 5, 6, 7, 8, and 24 h.

### 2.4 *C. elegans* cell dissociation protocol

The basic procedure described in this study is adopted from Zhang and co-workers (Zhang et al., 2011) but modifications were required to establish a cell dissociation protocol for application of the comet assay. As described above, nematodes were grown in the presence or absence of BaP until they reached the final larval stage. At this point, worms were washed off the plates with M9 buffer into 15 mL tubes and centrifuged at 1000*g* to form a pellet. The supernatant was discarded and replaced with fresh M9. Tubes containing nematodes were placed on a bench-top rotator for 3 min; thereafter worms were centrifuged at 1000*g* for 1 min to remove the bacteria. This step was repeated 10 times in order to flush the bacteria out of the nematodes’ gut. At the last wash, excess M9 buffer was removed leaving 1 mL of M9/nematode mixture and then transferred to 1.5-mL micro-centrifuge tubes. An aliquot of 40 μL sterile double deionized water was added to the worm pellet, and centrifuged at 16,000*g* for 2 min. After removal of the supernatant, 200 μL of fresh Triton X-100/sodium dodecyl sulfate (SDS)-dithiothreitol (DTT) solution (0.5% Triton X-100, 20 mM HEPES buffer, pH 8, 0.25% SDS, 200 mM DTT, and 3% sucrose) was added to the pellet. Worms were incubated at room temperature for 4 min immediately after which 800 μL of Egg buffer (25 mM HEPES, pH 7.3, 118 mM NaCl, 48 mM KCl, 2 mM CaCl_2_, 2 mM MgCl_2_) was added to each tube and centrifuged for 1 min at 16,000*g*. The supernatant was discarded and the worm pellet was washed 5 times with 1 mL of Egg buffer; the supernatant was removed after the last wash. An aliquot of 150 μL fresh papain (10 mg/mL; #3824, Applichem GmbH, Germany) was added to the tubes and worms were simultaneously mechanically disrupted by means of an electronic homogenizer (Ultra-Turrax T25; Janke & Kunkel, IKA-Labortechnik, Staufen, Germany). The homogenization time was typically 6 min (at ∼12,500 rpm). Total incubation time for each tube did not exceed 15 min (typically 8–12 min) to avoid damaging the cells. Thereafter, the enzymatic reaction was stopped by adding 1 mL of cold foetal bovine serum (#10106, Gibco, Fisher Scientific Ltd, UK) and the isolated suspension of nematode cells was centrifuged at 4 °C for 5 min at 9600*g*. The resulting pellet was washed 2 times with phosphate-buffered saline (PBS), followed by centrifugation at 4 °C for 5 min at 9600*g*. The pellet containing the isolated cells was re-suspended in 300 μL PBS and allowed to settle on ice for a period of 30 min. Next, the cell suspension was passed through a cell strainer (40 μm; #10737821, Fischer Scientific Ltd, UK) and the cell number in the eluate was adjusted in order to have a population of approximately 200 cells per slide for the comet assay.

### 2.5 Comet assay

The alkaline comet assay was conducted as described (Amaeze et al., 2015). Comets were analysed using a Leica fluorescence microscope (Leica DMLB 020-519-010 LB30T). DNA damage was scored using the Comet IV capture system (version 4.11; Perceptive Instruments, UK). Fifty cell nucleoids were assessed per slide, and each sample was analysed in duplicate. All samples were measured blind. The tail intensity (% tail DNA), defined as the percentage of DNA migrated from the head of the comet into the tail, was used as a measure of DNA damage induced, which is a meaningful end-point to assess genotoxicity (Forchhammer et al., 2012).

### 2.6 Statistical analysis

Statistical analysis was performed with Prism GraphPad Software (Version 6.04) and *P* < 0.05 was considered significant.

## 3 Results and discussion

One study previously aimed to utilise cultured embryonic cells of *C. elegans* to assess the DNA damaging effects of nicotine *in vitro* using the comet assay (Sobkowiak and Lesicki, 2009). More recently, Park et al. (2016) measured DNA strand breaks using the comet assay in mitotic germline nuclei of *C. elegans* after exposure to ionizing radiation. Whereas both these studies aimed to investigate DNA damage (*i.e.* comet formation) in a particular population of cells in *C. elegans* our approach focused to establish a protocol for cell dissociation from the whole nematode *C. elegans* to assess genotoxicity of environmental pollutants using the comet assay, thereby providing a measure of genotoxicity in the whole organism. The model compound we selected for study was the ubiquitous environmental pollutant and carcinogen BaP.

### 3.1 Bacterial growth analysis after BaP

**Fig. 1:**
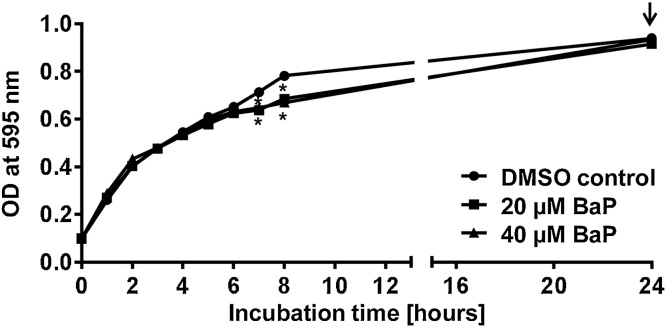
Bacterial growth curves of *E. coli* OP50 after BaP exposure for 24 h. Values are the mean ± SD (*n* = 3). Statistical analysis was performed by *t*-test and corrected for multiple comparisons using the Holm-Sidak method (*p < 0.05; different from DMSO control). Arrow indicates time-point at which nematodes are introduced (see Material and methods for details).

Because *C. elegans* are fed on *E. coli* OP50 it is essential to analyse the possible effects of BaP on bacterial growth (*i.e*. toxicity) before introducing the carcinogen-infused OP50 to the nematodes. OP50 growth was investigated over a period of 24 h after exposure to 20 and 40 μM BaP (Fig. 1). Over the course of 8 h growth of OP50 bacteria was significantly reduced after BaP exposure. However, overall differences in growth characteristics were relatively small and no longer apparent after 24 h of treatment, indicating no severe bacterial toxicity of BaP under these experimental conditions.

### 3.2 Effective disruption of the *C. elegans* nematodes to release cells and optimisation of the comet assay

**Fig. 2:**
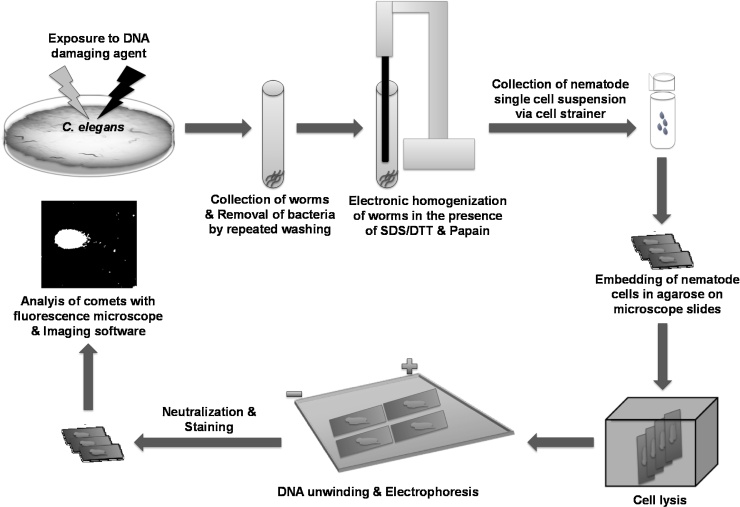
Scheme for the isolation procedure of cells from *C. elegans* for conducting the comet assay. See text for details.

To enable the use of the comet assay for DNA damage assessment in *C. elegans*, a method for cell dissociation from the nematodes was established (Fig. 2). The primary barrier to accessing cells and tissues in adult *C. elegans* is the cuticle. The anionic detergent SDS (0.25%) and 200 mM of the reducing agent DTT were used to dissolve the cuticle and release cells. Triton-X (1%) was added to the SDS-DTT solution as this improved cuticle degradation. Several mechanical and enzymatic approaches were subsequently examined in order to create a single-cell suspension from the nematodes. The tested proteases included pronase E and papain. Mechanical approaches included water bath sonication and the use of an electrical homogenizer. During the optimization process different protease concentrations, protease incubations times, sonication times, and times for homogenization were tested (data not shown). In the final protocol (see Material and methods) we used the protease papain in combination with simultaneous mechanic disruption by means of an electronic homogenizer (Fig. 2). It is important to point out that the timing established in the protocol is crucial to minimise cellular damage (*i.e*. background comet formation) in isolated cells from untreated nematodes. Before conducting the comet assay, any remaining nematode tissue debris was removed using a cell strainer. We noticed that the *E. coli* food source can result in high background on the comet assay slides. Therefore, nematodes were washed repeatedly with M9 buffer to remove the bacteria including those from the animal gut (see Material and methods).

### 3.3 Assessment of DNA damage of BaP in the comet assay

**Fig. 3:**
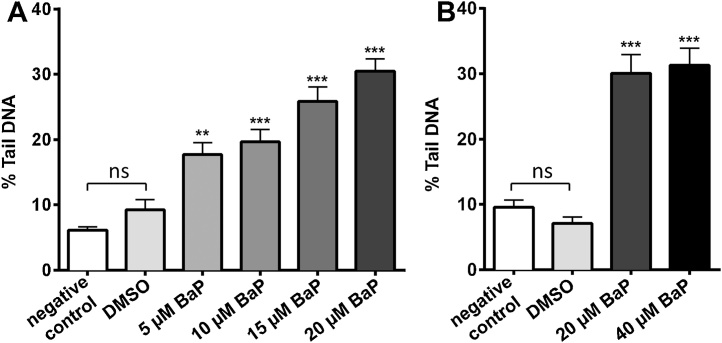
DNA damage as measured by the comet assay in wild-type *C. elegans* (N2 Bristol). (A) *C. elegans* nematodes were exposed to 0, 5, 10, 15 and 20 μM BaP from L1 to L4 stage for 48 h. (B) *C. elegans* nematodes were exposed to 0, 20 and 40 μM BaP from L1 to L4 stage for 48 h. Values represent mean ± SEM (*n* = 3). Statistical analysis was performed by one-way ANOVA followed by Tukey post hoc test [**p < 0.01, ***p < 0.001; different from DMSO control]; ns = not significant.

BaP-infused *E. coli* OP50 was provided to age-synchronised first larval stage (L1) wild-type nematodes allowing them to grow to the final larval stage (L4), typically 48 h. At this stage nematodes were collected and cells were isolated for DNA damage assessment in the comet assay. In the first experiment DNA damage (*i.e*. increase in% tail DNA) was evaluated using concentrations of 0, 5, 10, 15 and 20 μM BaP. We found that BaP-induced comet formation was dose-dependent (Fig. 3A). Concentrations as low as 5 μM BaP significantly induced DNA damage above background. At 20 μM BaP the DNA damage induced by BaP was ∼3-fold higher than controls (*i.e*. untreated). For controls we used nematodes given OP50 bacteria in the presence or absence of the solvent vehicle (*i.e*. DMSO). The background damage found in controls was ∼8-10% % tail DNA; DMSO treatment had no influence. Although most guidelines on the *in vivo* alkaline comet assay have been developed to assess rodent tissues, most of these studies consider background DNA damage of 5–15% tail DNA, depending of the tissue, as acceptable (Uno et al., 2015), which is in concordance with our findings. In a second experiment we tested 0, 20 and 40 μM BaP (Fig. 3B). Results at 20 μM BaP confirmed those obtained in the first experiment, indicating that the established protocol produces reliable results. Again, background damage observed in controls was ∼8–10% tail DNA. Furthermore we found that DNA damage induced by BaP reaches a plateau at 20 μM (∼30% tail DNA) with no further increase in comet formation at 40 μM BaP.

Although toxicity to PAHs such as BaP have been reported in the nematode *C. elegans* (Sese et al., 2009), most studies state that the *CYP1* family in humans does not have orthologs in *C. elegans* suggesting that nematodes lack the main CYP enzyme (*i.e*. CYP1A1) required for BaP activation (Leung et al., 2010). However, it was suggested that *cyp14A3* in *C. elegans* is a homolog of the human *CYP1A2* gene and is induced by BaP in *C. elegans* (Chakrapani et al., 2008). Others have shown that the *cyp35A* subfamily in *C. elegans* is induced by BaP concentrations as low as 1 μM (Menzel et al., 2001) suggesting that cyp35A may be able to metabolise BaP in *C. elegans*. The results of the present study clearly show that BaP causes DNA damage in nematodes detected in the comet assay (Fig. 3). Studies analysing the metabolic pathways of BaP in various invertebrates indicated that BaP-7,8-dihydrodiol, the major intermediate formed in mammals that is further metabolised by CYP1A1 and/or CYP1B1 to BaP-7,8-diol-9,10-epoxide (BPDE) (Arlt et al., 2015, Krais et al., 2016, Wohak et al., 2016), is not readily detectable in invertebrates (McElroy et al., 2000), suggesting that BaP may be activated also by CYP-independent pathways in nematodes (Saint-Denis et al., 1999). These pathways could include the generation of free radicals or the formation of reactive oxygen species leading to oxidative base modifications (Luch and Baird, 2005, Penning, 2014).

### 3.4 DNA damage assessment of BaP in DNA repair-deficient *C. elegans*

**Fig. 4:**
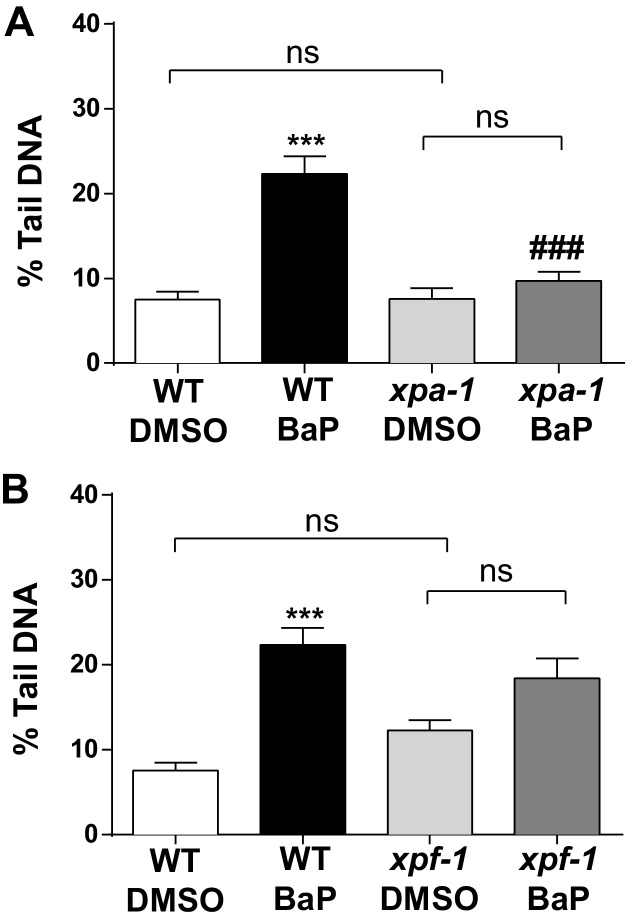
DNA damage as measured by the comet assay in wild-type (WT) *C. elegans* (N2 Bristol) and mutants *xpa-1* (A) and *xpf-1* (B). Nematodes were exposed to 15 μM BaP from L1 to L4 stage for 48 h. Values represent mean ± SEM (*n* = 3). Statistical analysis was performed by two-way ANOVA followed by Tukey post hoc test [***p < 0.001; different from wild-type DMSO control; ^###^*p* < 0.001; different from BaP-treated wild-type]; ns = not significant.

Many bulky DNA adducts (*e.g*. dG-*N*^2^-BPDE) are removed by nucleotide excision repair (NER) (Kucab et al., 2015, Kucab et al., 2016, Meier et al., 2014). BaP-induced DNA damage was evaluated in mutants harbouring deletions in genes involved in DNA repair (Fig. 4). Four mutant strains including *xpa-1*, *xpf-1, apn-1* and *exo-3* were investigated after exposure to 15 μM BaP and results compared to wild-type. XPA-1 (human XPA [Xeroderma Pigmentosum, complementation group A] related) and XPF-1 (Xeroderma Pigmentosum group F) are core NER factors. The endonucleases APN-1 and EXO-3 (ortholog of human APE1) in *C. elegans* are required for the repair of apurinic/apyrimidinic (AP) sites (Kato et al., 2015, Yang et al., 2012) which may be indicative of base excision repair (BER) activity.

#### 3.4.1 The impact of NER deficiency on baP-induced DNA damage *C. elegans*

DNA damage in BaP-treated *xpa-1* mutants diminished to background levels (Fig. 4A); BaP-induced comet formation was ∼2.5-fold lower compared to BaP-treated wild-type animals (∼22% tail DNA). These results suggest that due to the lack of NER function in *xpa-1* mutants bulky DNA adducts induced by BaP (*i.e*. dG-*N*^2^-BPDE adducts) are therefore not converted in NER-related single strand breaks which would be detected by the comet assay. We previously found that BaP failed to induce convincing levels of comet formation in human cells (Martin et al., 1999). However, BaP-induced comet formation markedly increased in the presence of DNA repair inhibitors indicating the importance of the DNA repair process in generating comets by BaP (Martin et al., 1999). In contrast, testing the activated metabolite of BaP, BPDE, in human cells, a recent study has questioned the importance of DNA excision repair activity in the removal of dG-*N*^2^-BPDE adducts as the cause of BPDE-induced DNA migration in the comet assay (Bausinger et al., 2016).

The effect of NER-deficiency in diminishing BaP-induced comet formation was less pronounced in *xpf-1* mutants than in *xpa-1* mutants (compare Figs. 4A and B). Comet formation in BaP-treated wild-type and BaP-treated *xpf-1* mutants was not significantly different (Fig. 4B). However, the induction of comets after BaP exposure increased only ∼1.4-fold in *xpf-1* mutants compared to ∼3-fold in wild-type relative to controls (*i.e*. untreated) (Fig. 4B).

Our conclusion that BaP forms bulky DNA adduct in *C. elegans* is in line with a previous study using extra-long PCR as an identifier of DNA adducts (Neher and Sturzenbaum, 2006). Contrasting results were found in another study (Leung et al., 2010) where exposure to 100 μM BaP did not cause any DNA damage that could be detected by qPCR in *C. elegans*. However, it should be noted that more sensitive methods are available to measure dG-*N*^2^-BPDE adducts in DNA using mass spectrometry or the ^32^P-postlabelling assay (Arlt et al., 2015, Krais et al., 2016, Wohak et al., 2016) which should be explored in future investigations.

#### 3.4.2 The impact of NER deficiency on baP-induced DNA damage *C. elegans*

**Fig. 5:**
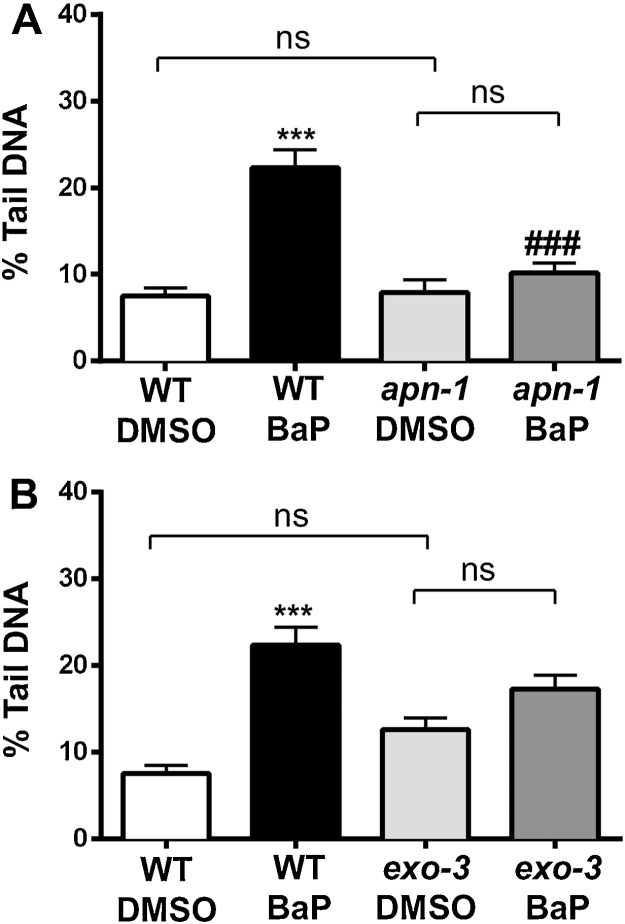
DNA damage as measured by the comet assay in wild-type (WT) *C. elegans* (N2 Bristol) and mutants *apn-1* (A) and *exo-3* (B). Nematodes were exposed to 15 μM BaP from L1 to L4 stage for 48 h. Values represent mean ± SEM (*n* = 3). Statistical analysis was performed by two-way ANOVA followed by Tukey post hoc test [***p < 0.001; different from wild-type DMSO control; ^###^*p* < 0.001; different from BaP-treated wild-type]; ns = not significant.

In BaP-treated *apn-1* mutants DNA damage did not increase above background levels (Fig. 5A). These results suggest that AP sites generated during BER after BaP-induced DNA damage are not readily converted into strand breaks due to the lack of AP endonuclease APN-1. APN-1 also possesses NER activity that recognizes and removes certain oxidized bases (Yang et al., 2012). Subsequently, knockout of APN-1 may also prevent the removal of oxidative base modification induced by BaP (*e.g*. 8-oxoguanine) which would be detected as DNA repair-related single strand breaks by the comet assay. Deletion of another endonuclease recognising AP sites, EXO3, had less impact on BaP-induced comet formation (Fig. 5B) suggesting that the distinct activities of APN-1 in *C. elegans* are not fully shared by EXO-3 (Kato et al., 2015).

## 4 Conclusion

We established a cell dissociation protocol of the whole nematode *C. elegans* that allows the assessment of genotoxicity of environmental pollutants using the single-cell electrophoresis assay (comet assay). We found that the ubiquitous environmental pollutant BaP induces comets in wild-type *C. elegans* in a dose-dependent manner and that transgenic *C. elegans* strains can be used to further characterise BaP-induced DNA damage pathways. Our results suggest that both NER- and BER-related single strand breaks contribute to comet formation induced by BaP. Future applications of the established protocol may include the modified comet assay using repair-specific endonucleases to detect oxidative damage to DNA. It can also be envisaged that reporter genes assays to evaluate transcriptional changes can be combined with the comet assay (as a measure of genotoxicity) in future investigations.

## Conflicts of interest

Nothing to disclose.
